# The Incidence of Liver Injury in Uyghur Patients Treated for TB in Xinjiang Uyghur Autonomous Region, China, and Its Association with Hepatic Enzyme Polymorphisms NAT2, CYP2E1, GSTM1 and GSTT1

**DOI:** 10.1371/journal.pone.0085905

**Published:** 2014-01-23

**Authors:** Yang Xiang, Long Ma, Weidong Wu, Wei Liu, Yongguang Li, Xia Zhu, Qian Wang, Jinfeng Ma, Mingqin Cao, Qian Wang, Xuemei Yao, Lei Yang, Atikaimu Wubuli, Corinne Merle, Paul Milligan, Ying Mao, Jiayi Gu, Xiumei Xin

**Affiliations:** 1 Department of Epidemiology and Biostatistics, School of Public Health, Xinjiang Medical University, Urumqi, Xinjiang, China; 2 The Red Cross of Xinjiang Uygur Autonomous Region, Urumqi, Xinjiang, China; 3 Center for Tuberculosis Control and Prevention, Xinjiang Uygur Autonomous Region Center for Disease Control and Prevention, Urumqi, Xinjiang, China; 4 Xinjiang Ili Kazak Autonomous Prefecture Centers for Disease Control and Prevention, Ili, Xinjiang, China; 5 Xinjiang Aksu District Center for Disease Control and Prevention, Aksu, Xinjiang, China; 6 Library of Xinjiang Medical University, Urumqi, Xinjiang, China; 7 Graduation School of Xinjiang Medical University, Urumqi, Xinjiang, China; 8 Faculty of Epidemiology and Population Health, London School of Hygiene and Tropical Medicine, London, United Kingdom; 9 Care Division, the Fifth Affiliated Hospital of Xinjiang Medical University, Urumqi, China; University of Cape Town, South Africa

## Abstract

**Background and Objective:**

Of three first-line anti-tuberculosis (anti-TB) drugs, isoniazid is most commonly associated with hepatotoxicity. Differences in INH-induced toxicity have been attributed to genetic variability at several loci, NAT2, CYP2E1, GSTM1and GSTT1, that code for drug-metabolizing enzymes. This study evaluated whether the polymorphisms in these enzymes were associated with an increased risk of anti-TB drug-induced hepatitis in patients and could potentially be used to identify patients at risk of liver injury.

**Methods and Design:**

In a cross-sectional study, 2244 tuberculosis patients were assessed two months after the start of treatment. Anti-TB drug-induced liver injury (ATLI) was defined as an ALT, AST or bilirubin value more than twice the upper limit of normal. NAT2, CYP2E1, GSTM1 and GSTT1 genotypes were determined using the PCR/ligase detection reaction assays.

**Results:**

2244 patients were evaluated, there were 89 cases of ATLI, a prevalence of 4% 9 patients (0.4%) had ALT levels more than 5 times the upper limit of normal. The prevalence of ATLI was greater among men than women, and there was a weak association with NAT2*5 genotypes, with ATLI more common among patients with the NAT2*5*CT genotype. The sensitivity of the CT genotype for identifying patients with ATLI was 42% and the positive predictive value 5.9%. CT ATLI was more common among slow acetylators (prevalence ratio 2.0 (95% CI 0.95,4.20) )compared to rapid acetylators. There was no evidence that ATLI was associated with CYP2E1 RsaIc1/c1genotype, CYP2E1 RsaIc1/c2 or c2/c2 genotypes, or GSTM1/GSTT1 null genotypes.

**Conclusions:**

In Xinjiang Uyghur TB patients, liver injury was associated with the genetic variant NAT2*5, however the genetic markers studied are unlikely to be useful for screening patients due to the low sensitivity and low positive predictive values for identifying persons at risk of liver injury.

## Introduction

China has a high incidence of tuberculosis (TB), ranking second in the world in terms of number of cases [1]. In Xinjiang, Northwestern China, the incidence of TB, the prevalence of smear positive TB and the TB mortality rate are higher than the national average and the Uyghur population are particularly at risk, but the prevalence of drug-induced liver injury, and associated risk factors, in this population have not been previously reported. The Directly Observed Treatment Short-course (DOTS), recommended by World Health Organization (WHO), constitutes the cornerstone of the current strategy for control of tuberculosis and covers the entire population of China. A combination of the three drugs, isoniazid (INH), rifampicin (RMP) and pyrazinamide (PZA) is used as the first-line therapy. However, multi-drug combination therapy is known to increase the risk of severe adverse drug reactions (ADRs) such as hepatotoxicity, gastrointestinal disorders, allergic reactions, arthralgia, neurological disorders, and other symptoms [2], [3]. Anti-TB drug-induced liver injury (ATLI) is the most prevalent and serious ADR encountered in the course of TB treatment [4]. In addition to the direct harm of ATLI, toxicity leads to early withdrawal from treatment, compromising the effectiveness of TB control (the China National Tuberculosis Prevention and Control Scheme (CNTS) [5]). The pathogenic mechanism of ATLI is still obscure [6]; a better understanding of the pathogenic mechanism may help to guide individual treatment and the implementation of the national anti-tuberculosis treatment program.

Anti-tuberculosis drug-induced hepatotoxicity is caused by the production of toxic metabolites of treatment drugs, associated with the presence of certain genetic variants of liver enzymes. These polymorphisms could potentially be used for screening patients and guiding their therapy. Among the first line anti-tuberculosis drugs, INH is more commonly associated with ATLI [7], [8], especially when used in combination with PZA. INH is metabolized to acetylisoniazid via hepatic N-acetyltransferase 2 (NAT-2) [8]. In turn, acetylisoniazid is hydrolyzed to acetylhydrazine, which is probably oxidized by cytochrome P4502E1(CYP2E1) to form some hepatotoxic intermediates [8], [9]. Direct hydrolysis of INH also generates hydrazine, a potent hepatotoxin. The hepatotoxins generated by NAT2 or CYP2E1 may be further detoxified by glutathione S-transferases (GSTs)(such as GSTM1and GSTT1) present in the liver [10]. Disposal of acetylhydrazine also depends on further acetylation by NAT2 to form a non-toxic metabolite, diacetylhydrazine [8]. Therefore, studies on genetic predisposition for ATLI have focused on a few metabolizing enzymes including NAT2, CYP2E1, GSTM1 and GSTT1. These enzymes are polymorphic, and the distribution of these polymorphisms varies among ethnic groups. Persons deficient in hepatic N-acetyltransferase may accumulate drugs that require acetylation. Individuals may be rapid, intermediate or slow acetylators, according to their activity of NAT2 [11], however the link between acetylator status and risk of ATLI has been inconsistent in previous studies [8], [12]–[22]. Inconsistent results have also been reported for the CYP2E1 polymorphisms [17], [18], [23]–[30]. Persons homozygous for GSTM1or the GSTT1null(non-functional) genotype, which cause lack of enzyme activity, have been found to be at increased risk of ATLI in some studies but again the results have been conflicting [17], [29], [31]–[38].

We assessed Uygur patients treated for TB in Xinjiang receiving standard short-course chemotherapy recommended by WHO, two months after they had started anti-TB treatment. We aimed to determine the prevalence of liver injury, and to investigate the association of genetic polymorphisms of NAT2, CYP2E1, GSTM1 and GSTT1 with hepatotoxicity, and their potential use for identifying patients at increased risk of liver injury.

## Methods and Design

### Patients

From January 2010 to May 2012, patients on TB treatment attending clinic two months after the start of treatment, were assessed for signs of liver injury, for risk factors for liver injury, and for genetic markers of liver enzyme polymorphisms. A total of 2244 newly diagnosed pulmonary TB patients belonging to the Uyghur ethnic group, from 12 counties in three regions of Xinjiang, who were receiving standard short-course chemotherapy recommended by WHO, who attended for a 2-month assessment, and any patients attending clinic with suspected liver disease after the start of treatment, prior to the 2-month visit, were invited to participate. Signed consent was sought after explaining the aims and procedures of the study. Patients who had signs of abnormal liver function when they started treatment (jaundice or elevated ALT,AST or bilirubin levels), or disease associated with liver dysfunction, were excluded. The study protocol was approved by the Ethics Committee of First Affiliated Hospital of Xinjiang Medical University.

All patients were prescribed INH(600 mg), RMP(600 mg, or 450 mg if the body weight was less than 50 kg), PZA (2,000 mg), and ethambutol (EMB) (1,250 mg) every other day in the first two months. After the two-month, INH and RMP were continued for a further four to six months. Retreatment patients in addition received streptomycin (SM) (750 mg) every other day in the first two months and continued receiving EMB for another six months. When patients developed a suspected Adverse Drug Reaction (ADR) (ATLI, gastrointestinal reaction, allergic reaction, nervous system disorders, or arthralgia), their treatment was adjusted according to the severity of the symptoms.

At the end of two months of anti-tuberculosis treatment, or if patients presented with symptoms suggestive of hepatitis (such as anorexia, nausea, vomiting, malaise) prior to the two-month visit, serum ALT, AST and total bilirubin levels were measured and a sample of each patient’s blood was stored for genotypic analysis. Patients were interviewed using a structured questionnaire and demographic information (gender, age, ethnicity), weight and height, TB treatment history, anti-tuberculosis treatment induced ADRs, clinical symptoms, and risk factors for liver disease, was recorded. Case notes of all suspected cases of ATLI were then reviewed and assessed by a clinician.

### Definition of ATLI

In the primary analysis ATLI was defined as an ALT, AST or bilirubin value more than two times the upper limit of normal value [39]. The upper limit of normal used in the study was 40 U/L for ALT, 40 U/L for AST, and 19µmol/L for total bilirubin. In secondary analyses we considered alternative case definitions, firstly with ATLI defined as values above three or above 5 times the upper limit of normal, and secondly defined as ATLI if patient ALT, AST or bilirubin levels corresponded to grade 3 or grade 4 adverse events in the The Division of AIDS table for grading the severity of adult and pediatric adverse events (http://rsc.tech-res.com/Document/safetyandpharmacovigilance/Table_for_Grading_Severity_of_Adult_Pediatric_Adverse_Events.pdf).

### DNA Preparation and Genotyping

Genomic DNA was extracted from^,^ peripheral blood leukocytes using the DNA isolation kit RelaxGene blood DNA DP319-02 (Tiangen, China) following the manufacturer's instructions and was stored at −20°C until used for genotyping, which was carried out by Shanghai BioWing Applied Biotechnology Company (http://www.biowing.com.cn).

In this study, we chose three NAT2 SNPs (SNP1rs1799929, SNP2 rs1799930 and SNP3 rs1799931) which have been shown to be associated with ATLI in adults treated with anti-tuberculosis drugs. All three SNPs of NAT2 were genotyped using the PCR/ligase detection reaction assay. The primers were 5′- CCT CTC CTGCAG GTGACC AT-3′and 5′- AGC ATGAAT CACTCT GCTTC-3′. Each set of ligase detection reaction probes comprised one common probe and two discriminating probes for the two types.

The target DNA sequences were amplified using a multiplex PCR method. PCR was performed in a final volume of 20 µl containing 1× PCR buffer, 3.0 mmol/l MgCl_2_,2.0 mmol/l deoxynucleotide triphosphates, 0.4 µl primers, 0.3 µl Qiagen HotStarTaq Polymerase (QIAGEN, China), 4 µl of 1×Q-solution, and 50 ng genomic DNA. Thermal cycling was performed in Gene Amp PCR system 9600 (PerkinElmer) with an initial denaturation of at 95°C for 15 min, followed by 35 cycles of denaturation at 94°C for 30 s, annealing at 62°C for 90 s, and extension at 64°C for 90 s, followed by a final extension at 64°C for 10 min.

The ligation reaction for each subject was carried out in a final volume of 10 µl containing 1× NEB Taq DNA ligase buffer, 12.5 pmol of each probe mix, 0.05 µl Taq DNA ligase[NEB Biotechnology (Beijing)], and 1 µl of multi-PCR product. A total of 35 cycles for ligase detection reaction was performed using 35 cycles at 95°C for 2 min, 94°C for 30 s, and 50°C for 2 min. The fluorescent products of ligase detection reaction were differentiated by ABI sequencer 377 (ABI).

Individuals were classified into three groups as follows: rapid acetylators (RA: a homozygote of NAT2*4), an intermediate acetylator genotype (IA: a heterozygote of NAT2*4 and mutant alleles) and a slow acetylator genotype (SA: a combination of mutant alleles), as in the Table 1.

**Table 1:** Definition of acetylator status according to NAT2 genotypes.

NAT2*5	NAT2*6	NAT2*7	Acetylator genotypes	Acetylator status
(rs1799929)	(rs1799930)	(rs1799931)		
C C	G G	G G	NAT2*4/4	RA
C T	G G	G G	NAT2*4/5	IA
C C	A G	G G	NAT2*4/6	IA
C C	G G	A G	NAT2*4/7	IA
T T	G G	G G	NAT2*5/5	SA
C T	A G	G G	NAT2*5/6	SA
C T	G G	A G	NAT2*5/7	SA
C C	A A	G G	NAT2*6/6	SA
C C	A G	A G	NAT2*6/7	SA
C C	G G	A A	NAT2*7/7	SA

For the Rsal SNP rs2031920 genotyping of CYP2E1, the method was the same as described above for NAT2. The primers were 5 ′-TTCATTCTGTCTTCTAACTGG-3′ and 5′-CCAGTCGAGTCGACATTGTCA- 3′.

In order to determine the presence or absence of the GSTM1 and GSTT1 genes, the multiplex PCR method was used, as described above. PCR conditions were 95°C for 15 min, followed by 35 cycles of 94°C for 30 s, 65°C for 90 s, 64°C for 90 s, and then a final extension step at 64°C for 10 min. The PCR products were resolved on ethidium bromide-stained 3% agarose gel. The primers used were F: CTCAGAGTTTCTGGGGAAGC and R: TGAGGGTAGAGAGGATATCTGATAGCA for GSTM1; F: GTTGCTCGAGGACAAGTTCC and R: ATCTGATTTGGGGACCACAG for GSTT1; and AGCTGCACTGTGACAAGCTG and TCATTCGTCTGTTTCCCATTC for β-globin gene.

### Statistical Methods

The primary analysis employed a definition of ATLI as ALT, AST or bilirubin more than twice the upper limit of normal, in a secondary analysis, ATLI was defined using a threshold of three times the upper limit of normal. Association analyses were done after excluding 297 individuals who had elevated levels of ALT, AST or bilirubin but did not meet the criteria for ATLI that could not be confidently ascribed as cases or non-cases, and individuals with missing genotype data. Association of ATLI with genotypes was assessed with the Cochrane-Armitage test and for acetylator status was assessed using a test for trend. 95% confidence intervals for the prevalence ratio were calculated using standard methods. All statistical analyses were conducted using SPSS software version 17.0 (SPSS, Chicago, IL) or Stata version 12 (College Station, Texas). The chi-square test was used to test for Hardy–Weinberg equilibrium.

## Results

### Characteristics of Patients and Incidence of ATLI

A total of 2244 Uyghur patients who had completed 2-months follow-up were assessed. 1858 (83%) had normal levels of AST, ALT and bilirubin; 297 (13%) had elevated levels but did not met the criteria for ATLI, and 89 patients (4%) were diagnosed with ATLI. Among these 89 cases, 74 had an ALT level above two times the upper limit of normal, and 15 patients had AST or total bilirubin above twice the upper limit of normal. 58/89 patients ALT, AST or bilirubin had two to three times of ULN (47 in ALT and 11 in AST/total bilirubin), 22/89 patients had levels three to five times of ULN (18 in ALT and 4 in AST/total bilirubin) and 9/89 had more than five times of ULN (all in ALT). Fifty-nine of the ATLI patients reported clinical symptoms (nausea, vomiting, anorexia or malaise). The baseline characteristics, dosages and liver function test results of the patient are shown in Table 2. The overall prevalence of ATLI was 4%, with increased risk among males, and in the south of Xinjiang compared to the north (Table 3).

**Table 2:** Characteristics of patients.

	Patients with ATLI	Patients with elevated	Patients with normal
		ALT,AST, or bilrubin	ALT, AST, bilirubin
Mean age in years (range)	37 (24–56)	43 (26–60)	46 (27–61)
BMI (kg/m2)	20.3 (18.4–22.6)	21.0(19.3–22.7)	21.4 (19.5–22.9)
INH (mg/kg/day)	4.8 (4.6–5.4)	5.2 (4.7–5.8)	5.4 (4.7–6.0)
PZA (mg/kg/day)	16.1 (15.2–17.9)	17.2 (15.6–19.2)	17.9 (15.6–20.0)
Liver function tests at 2 months:			
ALT	110.0 (84.3–147.0)	45.6 (37.5–54.8)	22.2 (16.8–29.0)
AST	91.4 (66.2–114.0)	42.5 (35.3–51.0)	23.0 (17.0–30.0)
bilirubin (µmol/L)	9.7 (5.1–16.5)	11.0 (6.7–14.0)	9.7 (6.0–12.5)

**Table 3:** Incidence of ATLI in patients tested at 2 months.

		PatientsWithATLI^1^	Patients withelevated ALT,AST, or bilrubin^2^	Patients with normalALT, AST andbilirubin levels^3^	Total	PrevalenceofATLI %	Prevalenceratio(95% CI)	P-value
Region	North Xinjiang	17	98	525	640	2.70%	1	
	South Xinjiang	72	199	1333	1604	4.50%	1.7	0.0446
							(1.00,2.80)	
Gender	Female	29	110	846	985	2.90%	1	
	Male	60	187	1012	1259	4.80%	1.6	0.0282
							(1.00,2.50)	
Treatment	Re-treatment	4	32	164	196	2.00%	1	
history	Primary	85	265	1694	2044	4.20%	2	0.1471
							(0.76,5.50)	
Age in years	<40years	52	136	803	991	5.20%	1	
	40–59years	18	86	537	641	2.80%	0.54	0.0175
							(0.32,0.91)	
	60+years	19	75	518	612	3.10%	0.59	0.0428
							(0.35,0.99)	

1 ALT, AST or bilirubin more than twice the upper limit of the normal range.

2 ALT, AST or bilirubin above the upper limit of the normal range and less than twice the upper limit of normal.

3 ALT, AST and bilirubin within the normal range.

### NAT2 Polymorphism in Patients with and without ATLI

Genotype distributions of NAT2, CYP2E1 and GSTM1and GSTT1 variants, among individuals who had normal levels for liver function tests, agreed with the Hardy-Weinberg equilibrium. NAT2*5, NAT2*6 and NAT2*7 were successfully genotyped in 1458, 1451 and 1468 subjects respectively. The NAT2*5 genotypes were associated with ATLI, with an increased prevalence in persons with the CT genotype, compared to CC, with a prevalence ratio of 1.70, 95% CI 1.10–2.70, Table 4. The sensitivity of the CT genotype for predicting ATLI was 42% and the positive predictive value 5.9%. There was no evidence of an association of NAT2*6 or NAT2*7 genotypes with ATLI. There was weak evidence of an association between ATLI and NAT2 acetylator status, (P-value for trend 0.06), with a 2-fold increase in prevalence in the slow acetylator group compared to rapid acetylators (prevalence ratio 2.0 (95% CI 0.95,4.20), Table 5).

**Table 4:** Prevalence of ATLI according to N-acetyltransferase 2 (NAT2) genotype.

Genotype		PatientsWithATLI	Patients withelevated ALT,AST, or bilrubin	Patients with normalALT, AST andbilirubin levels	Total	PrevalenceofATLI %	Prevalenceratio(95% CI)	P-value#
NAT2*5 (rs1799929)								
	CC	39	158	928	1125	3.50%	1	0.0623
	CT	30	81	397	508	5.90%	1.7	
							(1.10,2.70)	
	TT	2	7	62	69	2.80%	0.81	
							(0.20,3.30)	
Allelefrequency	C	76.10%		81.20%				
	T	23.90%		18.80%				
NAT2*6 (rs1799930)								
	GG	35	124	801	960	3.60%	1	0.4016
	GA	30	98	465	593	5.10%	1.4	
							(0.86,2.20)	
	AA	6	21	114	141	4.30%	1.2	
							(0.50,2.70)	
Allelefrequency	G	70.40%		74.90%				
	A	29.60%		25.10%				
NAT2*7 (rs1799931)								
	GG	58	198	1103	1359	4.30%	1	0.8623
	GA	12	45	274	331	3.60%	0.85	
							(0.46,1.60)	
	AA	1	0	20	21	4.80%	1.1	
							(0.16,7.70)	
Allelefrequency	G	90.10%		88.80%				
	A	9.90%		11.20%				
not typed		18		461	479	3.80%		
TOTAL		89	297	1858	224	4.00%		

# Cochrane-Armitage test.

**Table 5:** Prevalence of ATLI in patients according to acetylator status.

Acetylator status:	PatientsWithATLI	Patients withelevated ALT,AST, or bilrubin	Patients with normalALT, AST andbilirubin levels	Total	PrevalenceofATLI %	Prevalenceratio(95% CI)	P-value#
Rapid (RA)	9	41	287	337	2.70%	1	0.0616
Intermediate (IA)	34	118	667	819	4.20%	1.60 (0.75,3.20)	
Slow (SA)	28	79	422	529	5.30%	2.00 (0.95,4.20)	
not typed	18	59	482	559	3.20%		
TOTAL	89	297	1858	2244	4.00%		

# Cochrane-Armitage test for trend.

Among the slow-acetylator genotypes, the NAT2?5/6 genotypes was most strongly associated with ATLI (Table 6), sensitivity 25% and positive predictive value 7.5%.

**Table 6:** Prevalence of ATLI by NAT genotype.

Acetylatorstatus	Genotype	PatientsWithATLI	Patients withelevated ALT,AST, or bilrubin	Patients with normalALT, AST andbilirubin levels	Total	PrevalenceofATLI %	Prevalenceratio(95% CI)
Rapid	NAT2*4/4	9	41	287	337	2.7%	1.00
Intermediate	NAT2*4/5	12	41	231	284	4.2%	1.58 (0.68,3.70)
	NAT2*4/6	16	54	285	355	4.5%	1.69 (0.76,3.80)
	NAT2*4/7	6	23	151	180	3.3%	1.25 (0.48,3.30)
Slow	NAT2*5/5	2	7	62	71	2.8%	1.05 (0.25,4.50)
	NAT2*5/6	13	31	113	157	8.3%	3.10 (1.20,8.00)
	NAT2*5/7	5	8	51	64	7.8%	2.93 (0.59,15.00)
	NAT2*6/6	6	20	111	137	4.4%	1.64 (0.64,4.20)
	NAT2*6/7	1	13	67	81	1.2%	0.46 (0.06,3.90)
	NAT2*7/7	1	0	18	19	5.3%	1.97 (0.25,15.00)
	not typed	18	59	482	559	3.2%	
	TOTAL	89	297	1858	2244	4.0%	

### CYP2E1and GSTs Polymorphism in Patients with and without ATLI

The C1/C1 genotype of the CYP2E1 gene was most common in both the study groups. Only 1.9% of non-ATLI patients were found to carry the C2/C2 genotype of the CYP2E1 gene, and no ATLI case was found with the C2/C2 genotype (Table 7). There was no evidence of an association of the CYP2E1 Rsa?polymorphism, or the single or combined null GSTs genotypes, with ATLI.

**Table 7:** Genetic polymorphisms of CYP2E1,GSTM1 and GSTT1 and the prevalence of ATLI.

Variant		PatientsWithATLI	Patients withelevated ALT,AST, or bilrubin	Patients with normalALT, AST andbilirubin levels	PrevalenceofATLI %	Prevalenceratio(95% CI)	P-value*
CYP2E1 Rsa I	C1/C1	58	198	1066	4.40%	1	0.4708
(rs2031920):	C1/C2	12	41	267	3.80%	0.85(0.46,1.57 )	
	C2/C2	0	2	26	0.00%	0	
	not typed	19	56	499			
Allele frequency:	C1	91.40%		88.30%			
	C2	8.60%		11.70%			
GSTM:	GSTM1*1	34	116	669	4.20%	1	0.527
	GSTM1null	41	133	792	4.20%	1.02 (0.66,1.60)	
	not typed	14	48	397			
GSTT:	GSTT1*1	57	193	1041	4.40%	1	0.4627
	GSTT1null	18	57	420	3.60%	0.82 (0.49,1.40)	
	not typed	14	47	397			
GSTM1/GSTT1:	M1*1/T1*1	23	89	502	3.70%	1	0.2289
	M1*1/T1null	11	27	167	5.40%	1.40 (0.71,2.90)	
	M1null/T1*1	34	103	539	5.00%	1.30 (0.69,2.60)	
	M1null/T1null	7	30	253	2.40%	0.64 (0.29,1.40)	

* Cochrane-Armitage test.

## Discussion

Xinjiang ranks second highest in terms of the prevalence of TB and fifth highest prevalence of HIV of provinces in China. Xinjiang is an ethnically diverse region with Uyghur people forming 46% of the population. This is the first study to investigate the incidence of and risk factors for hepatic injury in TB patients in the Uhygur population. A better understanding of the risk factors and mechanisms of drug-induced hepatotoxicity may help to prevention this iatrogenic hepatic injury, which can be fatal. The study in over 2000 patients found a high incidence of elevated liver enzymes with 4% of patients with signs of liver injury after two months of treatment. Incidence was higher among men than women, and was higher among younger patients (under 40yrs) than in older groups. The association with gender is different from that seen in other studies, we did not find any gender-related predisposing factors which might explain the gender difference. Some studies [27], [40], [41] have shown that the women are at an increased risk of developing drug-induced hepatotoxicity during treatment of TB, but most studies did not find an association with gender [17], [18], [23], [25], [26], [28], [29], [32], [34], [36], [42], [43].

Acetylator status was associated with drug-induced hepatotoxicity, as has been reported in a number of previous studies [16], [18], [20]–[22], [24]–[26], [28], [44]–[47]. The genetic variant of NAT2*5 was associated with ATLI, in contrast to results from two recent case-control studies in TB patients in the Chinese Han population [19], [31] which did not find an association of four common SNPs with ATLI. However the association was weak and the sensitivity of the markers studied for identifying persons with ATLI was low. We employed the same case definition for ATLI used in a number of previous studies. When more restrictive definitions were used, the number of cases was reduced but the predictive value of the genetic markers remained low.

A limitation of the present study is that although before starting anti-tuberculosis therapy, it is normal practice for liver and renal function to be tested, it was not possible to confirm that these tests were performed it is therefore the possibility that some patients had signs of liver disease that were missed when treatment started cannot be ruled out, we did not have detailed information about adherence, and there may be residual confounding due to uncontrolled risk factors for liver disease, although alcohol consumption is unlikely to be an important factor in this muslim population. In addition, other plausible candidate genes associated with the ATLI need to be explored [48].

The disposition of anti-tuberculosis drug is related to the activity of many drug-metabolizing enzymes (DMEs), including NAT2, CYP2E1and GST. Polymorphisms of some encoding genes may influence the activity of the corresponding DMEs. In view of NAT2*5(C481T, rs1799929), NAT2*6 (G590A, rs1799930)and NAT2*7(G857A, rs1799931) mutations accounted for virtually all of the slow acetylator alleles in Asians, we have evaluated the roles of NAT2*5, NAT2*6 and NAT2*7 in the developing of ATLI basing on our community-based TB patients. The present study did not find NAT2*6 and NAT2*7 were risk for developing ATLI,while other studies [18], [44], [45], [49] have reported NAT2*6 and NAT2*7 had a higher incidence of drug-induced hepatotoxicity. However, the association of NAT2*5 and ATLI observed in our study population, agrees with findings of Possuelo LG *et al.*, in southern Brazil [44].

Acetylation activity in vitro is ranked in the sequence:NAT2*4>NAT2*7>NAT2*6>NAT2*5 [16], [46]. The NAT2*5 mutant homogeneous genotype has not been reported in some previous studies [19], [47], [49], but in Uyghur patients NAT2*5 is the predominant allele with low acetylation activity. Slow acetylators homozygous for the NAT2*5 allele may acetylate isoniazid more slowly. This is in line with our finding that slow acetylators with NAT2*5/6 has a significantly higher risk of anti-tuberculosis drug-induced hepatitis than other genotypes.

These results may reflect ethnic differences. The proportion of rapid, intermediate and slow acetylators in this study (21%, 49% and 31% respectively) differs from that reported for the healthy Chinese population [50] (30%,45% and 25%) and from other results [19]. This may be due to the presence of other polymorphisms in the NAT2 gene. Studying other polymorphisms, such as NAT2*12 and NAT2*13, may help to clarify these conflicting results.

Among several CYP2E1 genetic polymorphisms, the RsaI polymorphism has been evaluated mostly in association with ATLI, explained by a higher CYP2E1 activity with the c1/c1 genotype and the inhibitory effect of INH [17], [26], [29], [45], [51]. Some studies have shown the relationship between the CYP2E1 RsaI c1/c1genotype and the risk of anti-TB drug-induced hepatitis [17], [23], [26], [43], [51], [52].

Huang et al. [23] in a Taiwanese population demonstrated that the CYP2E1 c1/c1 genotype increased the risk of anti-TB drug-induced hepatotoxicity, and similar results have been found in China [17]. Other studies found conflicting results [18], [24], [25], [27]–[31], [53]. Two recent studies in China showed no evidence of association between CYP2E1 c1/c1 genotype and ATLI [24], [31]. In our study, the CYP2E1 mutant genotype was rare and therefore unlikely to be useful as a predictor of ATLI risk.

GST, which plays an important protective role in preventing ATLI, exists in several isoforms. [10], [54] Some genetic loci, notably GSTM1 and GSTT1, are polymorphic. Homozygous ‘null’ mutations result in a complete absence of enzyme activity. It is speculated that people with null GSTM1 or GSTT1 genotypes could not detoxify the toxic reactive metabolites efficiently, and thus have higher risk of ATLI. Some studies have reported a relationship between GSTM1 null [17], [29]–[38] and GSTT1[29]–[38] null genotype, and ATLI. However, most of these studies [29]–[34] found no evidence of an association, including studies in the Chinese population.

According to the model of the pathogenesis of drug-induced hepatotoxicity proposed by Russmann *et al.*, the biochemical mechanism of drug-induced hepatotoxicity may involve a complex interplay between the chemical properties of the drug, environmental factors (such as age, sex, diet, alcohol consumption, compliance of drug intake, existing liver disease, concomitant use of other drugs and comorbid illness) and genetic factors that control the handling of the drug (metabolism, detoxification, and transport), as well as those that influence cell injury and repair [55]. The development of pharmacogenomics enables more extensive studies using larger sample sizes and high-throughput DNA microarrays, which may include more genotypes of relevant DMEs [15]. Most previous studies have been based on the INH metabolic pathway [56], but INH, RMP and PZA are all hepatotoxic drugs [57] and different may have different pathogenic mechanisms [58]. Other factors may influence gene expression transcription and translation of CYP2E1. Gene methylation may influence the expression of CYP [59]. Vieira et al. [60] reported an association between CYP2E1 transcripts and decreased methylation of CpG residues in intron 1 of the CYP2E1gene, during the late neonatal period. In addition microRNA (miRNA) regulation of CYP has been described [59]. Human CYP2E1 expression is regulated by miR-378, mainly via translational repression [61]. Some studies indicate that a mutant C allele of manganese superoxide dismutase, the absence of HLA-DQA1*0102 and the presence of HLA-DQA1*0102 alleles, may be associated with ATLI [37], [62]. The biochemical mechanism and pathogenesis of ATLI is complex and multifactorial, it may be valuable to combine several risk factors to identify patients at increased risk of ATLI.
